# In vitro anticancer activity of ethanol extract of *Adhatoda vasica* Nees on human ovarian cancer cell lines

**DOI:** 10.1186/s43141-021-00215-1

**Published:** 2021-08-05

**Authors:** J. N. Nikhitha, K. S. Swathy, R. Pratap Chandran

**Affiliations:** 1 Department of Biotechnology and Research, K. V. M. College of Science and Technology, Kokkothamangalam P.O., Cherthala 688527, Alappuzha District, Indore, Kerala State India; 2 Department of Biotechnology, Indhira Gandhi College of Arts and Science, Nellikuzhi, Kothamangalam, Ernakulam District, Indore, Kerala State India

**Keywords:** *Adhatoda vasica*, Anticancer, Cell line, Ovarian cancer, Teratocarcinoma

## Abstract

**Background:**

Ovarian cancer causes more deaths than any other cancer of the female reproductive system because there is no effective screening and most women are diagnosed at advanced stages. The probability of survival at 5 years is less than 30%, and the limitation is that it will not respond to chemotherapy protocol and surgery as well. Moreover, some evidence have shown potential anticancer properties of flavonoids, protective chemicals in plant foods, such as being an antioxidant, antiestrogenic, antiproliferative, and antiinflammatory. In this study, the anticancer activity of crude ethanol extracts of leaves from *Adhatoda vasica* was investigated.

**Results:**

By the application of a cell-based assay, the LC 50 value of the *A. vasica* which showed anticancer effect was used for further studies. The cell line treated with LD 50 value of *A. vasica* extracts was observed for 0 h, 24 h, and 48 h to reveal the inhibition of the metastatic property in treated PA1 cells. The mRNA isolated from the teratocarcinoma PA1 cells treated with the *A. vasica* extract was further converted to cDNA and was amplified for the analysis of the p53 gene, p21 gene, and GAPDH gene expression. The expression in treated cells and the untreated control indicated the activity of the *A. vasica* extract against the ovarian cancer.

**Conclusion:**

The present study suggested the antiproliferative and antimetastatic effects of medicinal plant *A. vasica* on PA1 cells.

## Background

Ovarian carcinoma could originate from any of three potential sites: the surfaces of the ovary, the fallopian tube, or the mesothelium-lined peritoneal cavity. During initial tumorigenesis, ovarian malignant cells undergo an epithelial to mesenchyme transition, which involves a change in cadherin and integrin (marker) expression and up-regulation of photolytic pathways [1, 2]. The initial steps of metastasis are regulated by a controlled interaction of adhesion receptors and proteases, while late metastasis is characterized by the oncogene-driven fast growth of tumor. The prolonged use of platinum-based chemotherapy often leads to drug resistance, which causes the ovarian cancer patient to relapse and potential death [3, 4]. Therefore, there is an urgent medical need for breakthrough drugs with an effective therapeutic impact on ovarian cancer [5]. Since phytochemicals have been used over years as treatment for various diseases, because of their huge chemical diversity and wide range of biological activity, subsequently around 60% of the currently used anticancer drugs were derived from natural sources including plants [6, 7]. Hence, evolved the rationale of treating cancer using secondary metabolites isolated from the plant extracts with the most effective mechanisms that enhance P53 protein expression, reducing the expression of proteins P27, P21, and NFκB and induction of apoptosis, inhibition of the PI3K/Akt pathway, and reduction of the level of acid phosphatase and lipid peroxidation of herbal plants that can inhibit cell cycle and proliferation [8, 9].

*Adhatoda vasica* belongs to the family Acanthaceae, commonly known as Adosa, which is found in many regions of India. It has a multitude of uses in traditional Ayurveda. *A. vasica* is most well known for its effectiveness in cancer treatment. The leaf of *A. vasica* showed a stimulant effect on the cancer cells. *A. vasica* shows many antispasmodic and expectorant effects and is also used for the treatment of asthma, bronchitis, and other respiratory conditions [10]. The prominent alkaloid found in *A. vasica* leaf is the quinazoline alkaloid known as vasicine. In addition to vasicine, the leaves and roots of *A. vasica* contain the alkaloids l-vasicinone, deoxyvasicine, mainontone, vasicinolone, and vascinol [11]. Alkaloids play a major role as anticancer agents by inhibiting the enzyme topoisomerase which is involved in DNA replication, inducing apoptosis and expression of the P^53^ gene. The development of novel strategies to re-activate mutant p53 is required to provide clues to effectively treat malignant cancers bearing p53 mutations [12].

The cyclin-dependent kinase inhibitor p21 (also known as p21^WAF1/Cip1^) promotes cell cycle arrest in response to many stimuli, served as both a sensor and an effector of multiple antiproliferative signals. The p21 functions as a mediator of p53 tumor suppressor activity inhibiting cell cycle progression through its inhibitory effect on cyclin-dependent kinase (CDK) cyclin complexes and proliferating cell nuclear antigen (PCNA). The tumor suppressor activity of p21 stemmed from inducing growth arrest, differentiation or senescence, and DNA repair [13, 14]. Recently, it has become apparent that p21 can be stimulated by other pathways independent of p53. p21 directly regulates gene expression and other cellular events through protein–protein interactions which are independent of CDKs and PCNA. Recent data suggest a tumorigenic role of p21 in certain contexts relying on its ability to suppress human teratocarcinoma cell line apoptosis and promote the assembly of type-D cyclins with CDK4 and CDK6 [15]. Given that p21 is a tumor suppressor, yet behaving as an oncogene in certain cellular contexts, therefore, targeting p21 or factors regulating its activity for therapeutic intervention may be a promising but challenging task [16, 17]. The present study was designed to investigate the anticancer activity of *A. vasica* ethanol leaf extract against PAI cell lines [18, 19].

## Methods

*A. vasica* leaf samples were collected from Poojappura, Thiruvananthapuram, Kerala state, India. This plant material was identified by Dr. Shaji P.K., Scientist, Environmental Resources Research Centre, P.B. No. 1230, P.O. Peroorkada, Thiruvananthapuram, Kerala state, India. All chemicals used in the study were of analytical grade and obtained from Invitrogen, Sigma, and HiMedia Laboratories Private Limited, Mumbai, India. The cell line used in the study was PA 1 (human teratocarcinoma) cell lines which were procured from the National Centre for Cell Science (NCCS), Pune, Maharashtra, India.

### Preparation of crude extracts

*A. vasica* leaf was carefully plucked and washed. The washed leaves were air dried and chopped into small pieces. As the leaves get well dried, the leaves get weighed. Twenty grams of dried leaves was then treated with 70% ethanol kept in a shaker for 24 h. The leaves were squeezed out and air dried. Then, the dried extract was used as the test sample.

### Test for alkaloids

#### Dragendroff’s test

0.25 ml of Dragendroff’s reagent was added to the previous mixture for precipitation, and the precipitate was centrifuged over 5 min at 3000 rpm and then further washed with 0.25 ml of ethanol. The filtrate was discarded and the residue was then treated with 0.25 ml of disodium solution (1% w/v). The brownish black precipitate formed was then centrifuged for 5 min at 3000 rpm. This residue was dissolved in 0.2 ml of concentrated nitric acid, and 0.1 ml was then pipetted out and mixed with 0.5 ml of thiourea solution (3% w/v). The absorbance of this solution was measured at 435 nm using UV–visible spectrophotometer (Agilent, Cary 60) against a blank containing 0.1 ml of concentrated nitric acid and 0.25 ml of thiourea solution (3% w/v), and the values obtained were interpreted using the standard graph of caffeine to get the milligram equivalents of caffeine [20].

### Anticancer effect of *A. vasica* extract against PA 1 cell lines

PA 1 cell lines were grown in Dulbecco’s modified Eagle’s medium supplemented with fetal bovine serum (FBS), penicillin, and streptomycin. The cells were subcultured after trypsinization with 0.25% in 0.5 mM EDTA and were cultured under 5% CO_2_ at 37 °C.

### Dulbecco’s modified Eagle’s medium (DMEM) preparation

DMEM (0.67 g) was suspended in 25 ml tissue culture grade water with constant stirring until the powder was completely dissolved. The water should not be heated, and 0.187 g of NaHCO_3_ powder was added and stirred until dissolved. The pH was adjusted to 0.2, 0.3 pH units below the desired pH (pH −7.4) using 1 N HCl or 1 N NaOH since the pH tends to rise during filtration. The final volume was made up to 50 ml with tissue culture grade water. The medium was sterilized immediately by filtering through a sterile membrane filter with a porosity of 0.22 μm or less. Positive pressure was used rather than vacuumed to minimize the loss of CO_2_. Aseptically sterile supplements were added as required, and the desired amount of sterile medium was dispensed into sterile containers. The required medium was then stored at 2 to 8 °C in dark for further use.

### Trypsinization

It is the process of using trypsin, a proteolytic enzyme which breaks down proteins, to dissociate adhered cells from the vessels in which they were being cultured. The cell lines were washed with phosphate-buffered saline (PBS). Five hundred microliters of trypsin was added in cultured cell lines for 3 min at 37°C. After disaggregation, the cells were transferred to another flask and supplemented with media.

#### Subculturing

Subculturing involves transferring a small number of cells into a new vessel. A confluent plate was taken for subculturing. For subculturing, firstly, remove media from the flask. The flask was then washed twice with PBS and then 200 μl of trypsin (0.25% in 0.5 Mm EDTA) was added to the flask. Incubation was done for 3 min. Mixing was done properly with a pipette throughout the incubation time. Fresh media were added to the trypsinized cells and mixing was done. One hundred microliters of suspension was transferred to a 24-well plate. Fresh media were added to both the mother flask and the 24-well plate. It was then plated in a humified incubator at 37 °C with a 5% CO_2_ incubator.

### Sample addition

After attaining sufficient confluency, the cells were trypsinized (500 μl of 0.025% Trypsin in PBS/0.5 mM EDTA solution (Himedia Laboratories, India)) for 2 min and passaged to T flasks in complete aseptic condition. The cells were treated with a sample of different concentrations (100 μg, 50 μg, 25 μg, 12.5 μg, 6.25 μg in 500 μl of 5% DMEM) and were incubated for 24 h before the staining procedure starts.

### In vitro cytotoxic effect by sulforhodamine B colorimetric assay

#### Cell fixation and staining

In the well plate without removing the cell culture, supernatant, 100 μl of cold 10% trichloroacetic acid (TCA), was added and incubated at 4 °C for 1 h. After which, the plates were washed four times with slow running tap water and any excess water was removed using paper towels. The plate was dried using a blow dryer to completely dry them. Once the plates are dry, 100 μl of 0.057% sulforhodamine B (SRB) solution was added to each well. The stain was allowed to be incubated for 30 min followed by briefly rinsing the plate four times with 1% acetic acid to remove any excess unbound dye. This was followed by the addition of 200 μl of 10 mM Tris base solution (pH 10.5) to each well. The plates were then placed in a shaker for 5 min to solubilize the protein-bound dye. The OD was read in a microplate reader at 510 nm.

$$ \mathrm{Percentage}\ \mathrm{viability}\ \left(\%\right)=\frac{\mathrm{Absorbance}\ \mathrm{of}\ \mathrm{test}}{\mathrm{Absorbance}\ \mathrm{of}\ \mathrm{sample}}\times 100 $$

### Migration assay

PA1 (ovarian cancer) cell lines were maintained in Dulbecco’s modified Eagle’s medium (Gibco, Invitrogen). The cell line was cultured in a 25-cm tissue culture flask with DMEM supplemented with 10% FBS, l-glutamine, sodium bicarbonate, and antibiotic solution containing penicillin (100 U/ml), streptomycin (100 μg/ml), and amphotericin B (2.5 μg/ml). Cultured cell lines were kept at 37 °C in a humidified 5% CO_2_ incubator (NBS Eppendorf Germany).

Exponentially growing cells were trypsinized and seeded at a density of 200,000 cells per well into a 12-well plate for 24-h incubation (approximately 90% confluent). The scratch wounds were made by a sterile 1-ml pipette tip through a pre-marked line. After removal of the resulting debris from five lineal scratches, the cell monolayer was subsequently rinsed three times with PBS followed by incubation with LD 50 concentration of extracts for 0 h, 24 h, 48 h, and 72 h.

The wound areas were displayed by taking images just above the interchanges between scratched wound areas’ pre-marked lines, and the effect of the sample on wound closure was determined microscopically at 4× magnification (Olympus CKX41) after incubation. The effect of the sample on wound closure was measured in terms of the area using MRI-ImageJ analysis software.

#### Gene expression study

PA 1 cells were cultured in a 25-cm^2^ tissue culture flask with DMEM supplemented with 10% FBS, l-glutamine, sodium bicarbonate (Merck, Germany), and antibiotic solution containing penicillin (100 U/ml), streptomycin (100 μg/ml), and Amphotericin B (2.5 μg/ml). Cultured cell lines were added with LD 50 concentration of the sample and were kept at 37 °C in a humidified CO_2_ incubator (NBS Eppendorf, Germany). An untreated control was also maintained.

#### Isolation of total RNA

Total RNA was isolated using the total RNA isolation kit according to the manufacturer’s instruction (Invitrogen product code 10296010). The addition of TRIzol solution causes the disruption of cells and the release of RNA. Chloroform extraction was performed following centrifugation, exclusively in the aqueous phase, whereas proteins are in the interphase and organic phase. On mixing with isopropanol, RNA gets precipitated as a white pellet on the side and bottom of the test tube.

After attaining 70% confluency of cells in a 6-well plate (approximately 4 × 10^5^ cells), the cells were treated with samples and incubated for 24 h. A set of untreated control were also incubated at 37 °C for 24 h in a CO_2_ incubator. After incubation, DMEM media were removed aseptically and 200 μl of TRIzol reagent was added to the culture well plate and incubated for 5 min. The contents were then transferred to a fresh sterile Eppendorf tube. Two hundred microliters of chloroform was added and shaken vigorously for 15 s and incubated for 2–3 min at room temperature, followed by centrifugation at 1400 rpm for 15 min at 4 °C. The aqueous layer was collected and 500 μl of 100% isopropanol was added. It was incubated for 10 min at room temperature and then centrifuged at 1400 rpm for 15 min at 4 °C. The supernatant was discarded and the pellet thus obtained was washed with 200 μl of 75% ethanol. It was then centrifuged at 14000 rpm for 5 min at 4 °C in a cooling centrifuge (Remi CM12).

The RNA pellet was dried and suspended in TE buffer.

#### cDNA synthesis and amplification

The cDNA synthesis was performed using Thermo scientific verso cDNA Synthesis kit (Product code AB-1453/A). About 4 μl of 5X cDNA synthesis buffer, 2 μl of dNTP mix, 1 μl of anchored Oligo dT, 1 μl of RT Enhancer, 1 μl of Verso Enzyme Mix, and 5 μl of RNA template (1 mg of total RNA) were added to an RNAse-free tube. Then, the total reaction volume was made up to 20 μl with the addition of sterile distilled water. The solution was mixed by pipetting gently up and down. The thermal cycler (Eppendorf Master Cycler) was programmed to undergo cDNA synthesis. The cDNA synthesis was employed for 30 min at 42 °C and inactivation was employed for 2 min at 95 °C.

The amplification was done using the Thermostatic amplification kit. The following components were added to a new PCR vial in a PCR workstation: for each 50 μl reaction, 25 μl of PCR Master Mix (2X), 2 μl of forward primer (0.1–1.0 μM), 2 μl of reverse primer (0.1–1.0 μM), and 5 μl of template DNA (10 pg–1 μg). The components were made up to 50 μL with sterile distilled water (nuclease free). Initial denaturation was at 95 °C for 3 min, followed by denaturation at 95 °C for 30 s, annealing at Tm for 30s, and extension at 72 °C for 1 min which was repeated for 35 cycles and the final extension at 72 °C for 5 min. After the amplification, the PCR product was separated by agarose gel electrophoresis. The following are the forward sequence (5′–> 3′) and reverse sequence (5′–> 3′) used in the electrophoresis:

**Table:** 

Human bactin—TCACCCACACTGTGCCCATCTACGA(25)[Tm 66.3],	
CAGCGGAACCGCTCATTGCCAATGG(25) [Tm 67.9]	
Human p 21—GAGGCCGGATGAGTTGGGAGGAG(24)[Tm 69.6],	
CAGCCGGCGTTTGGAGTGGTAGAA(24 [Tm 66.1]	
Human p 53—CCCCTCCTGGCCCCTGTCATCTTC(24) [Tm 69.6],	
GCAGCGCCTCACAACCTCCGTCAT(24) [Tm 67.8]	

### Agarose gel electrophoresis

Agarose gel electrophoresis is a method for separating and visualizing DNA fragments. The fragments are separated by charge and size move through an agarose gel matrix, when subjected to an electric field. The electric field is generated by applying a potential across an electrolyte solution (buffer). When boiled in an aqueous buffer, agar dissolves and upon cooling solidifies to a gel. 1.5% agarose gel was prepared in 1x TE buffer and melted in a hot water bath at 90 °C. Then, the melted agarose was cooled down to 45 °C. Six microliters of 10 mg/ml of ethidium bromide was added and poured into a gel casting apparatus with the gel comb. After setting, the comb was removed from the gel. The electrophoresis buffer was poured into the gel tank and the platform with the gel was placed in it so as to immerse the gel. The gel was loaded with the samples and run at 50 V for 30 min. The stained gel was visualized using a gel documentation system (E gel imager, Invitrogen).

## Results

### Crude extracts

The extracts of the *A. vasica* plant were done using the cold percolation method using 70% ethanol as solvent and were kept in a shaker for 48–72 h. Afterwards, they were squeezed using a muslin cloth and the extract was kept for drying. The dried extract was used for the analysis in the whole study.

### Qualitative analysis

The presence of alkaloid was determined using Dragendroff’s method and the formation of orange red color indicated a positive alkaloid reaction.

### Quantitative analysis

**Fig. 1:**
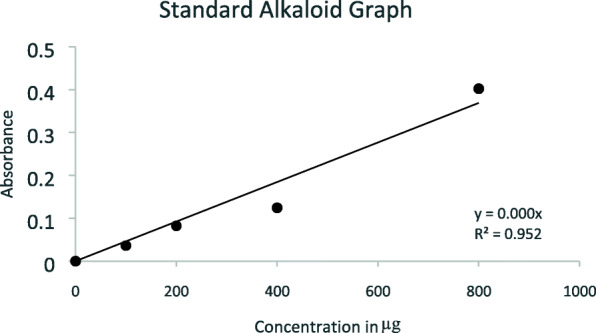
Alkaloid standard graph

The extract was further estimated for the determination of the amount of alkaloids present in it from a stock of 10 mg of extract in 1 ml DMSO, and 16.8 μg of alkaloid was estimated to be present in 1 mg of extract from the standard graph (Fig. 1).

### Sulforhodamine B test

The *A. vasica* extract was further treated with PA 1 cell line to check its anticancer effect. The anticancer ability was initially evaluated using SRB staining assay for the cells treated with different concentrations of the *A. vasica* extract, which were initially visualized under a phase contrast microscope for detecting the morphological changes seen in the cells treated with higher concentrations of the extract.

**Fig. 2:**
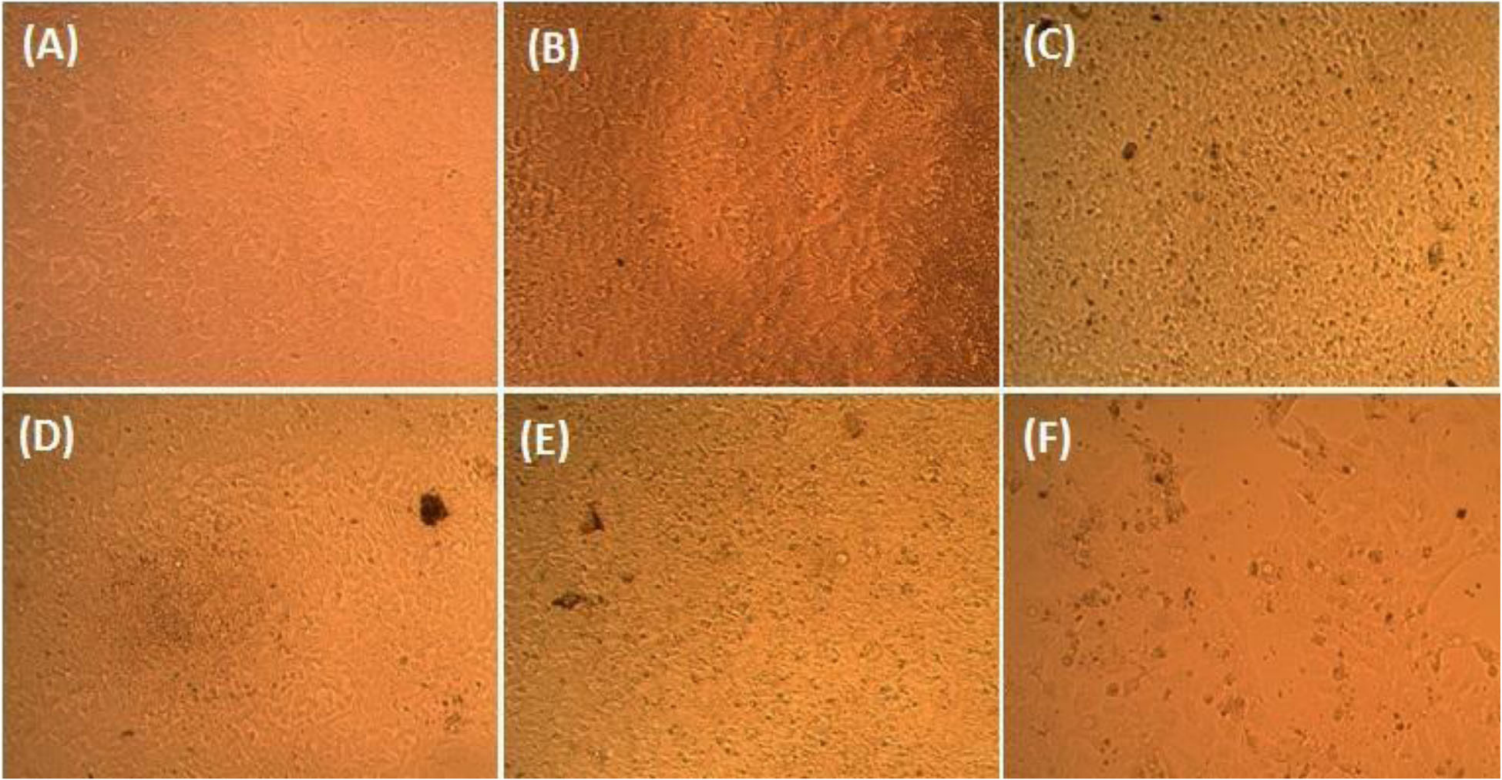
PA1 cells visualized under a phase contrast microscope. **A** Control, treated with different concentrations of the sample (in μg): **B** 6.25, **C** 12.5, **D** 25, **E** 50, and **F** 100

**Fig. 3:**
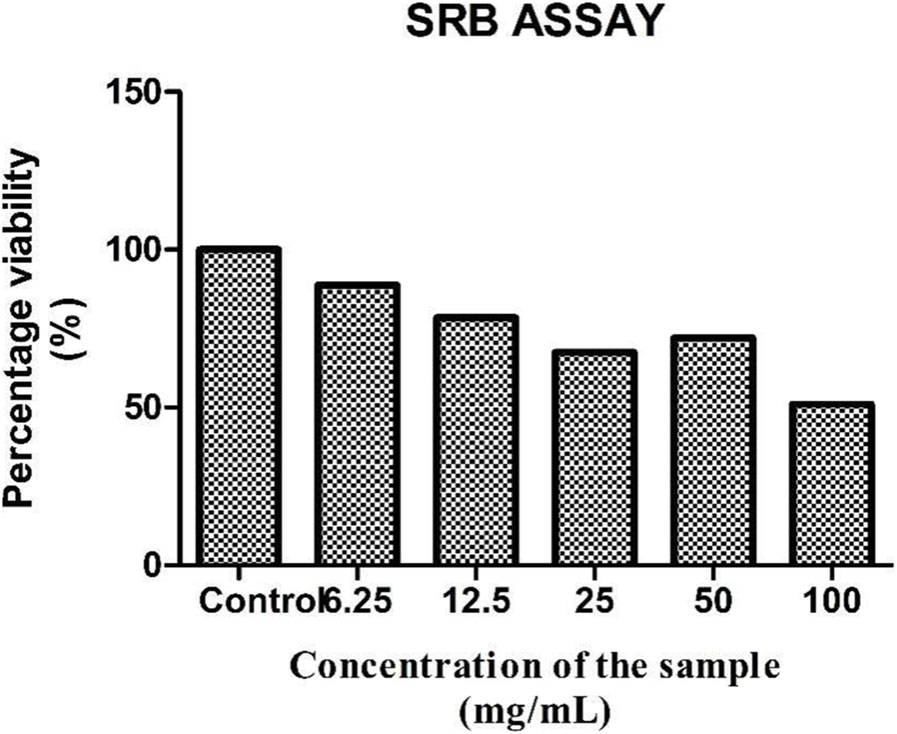
Anticancer activity exhibited by *Adhatoda* extract

The SRB assay was done to measure drug-induced cytotoxicity and cell proliferation for drug screening applications. From the results, it is observed that as the concentration of the *A. vasica* extract increased for treatment, the percentage viability of PA1 cancer cells decreased linearly. This observation might highlight the potential anticancer effect of the extract with a LC 50 value of 107.339 μg/ml (calculated using ED50 PLUS V1.0 Software as illustrated in Figs. 2 and 3). As seen in Fig. 2, a gradual decrease in the number of cells (B, C, D, E, and F) and also the morphological changes induced by the extract on PA1 cells such as membrane blebbing, cell shrinkage, etc. when compared to the untreated control (A) indicate the cytotoxic effect of the extract.

### Migration assay

**Fig. 4:**
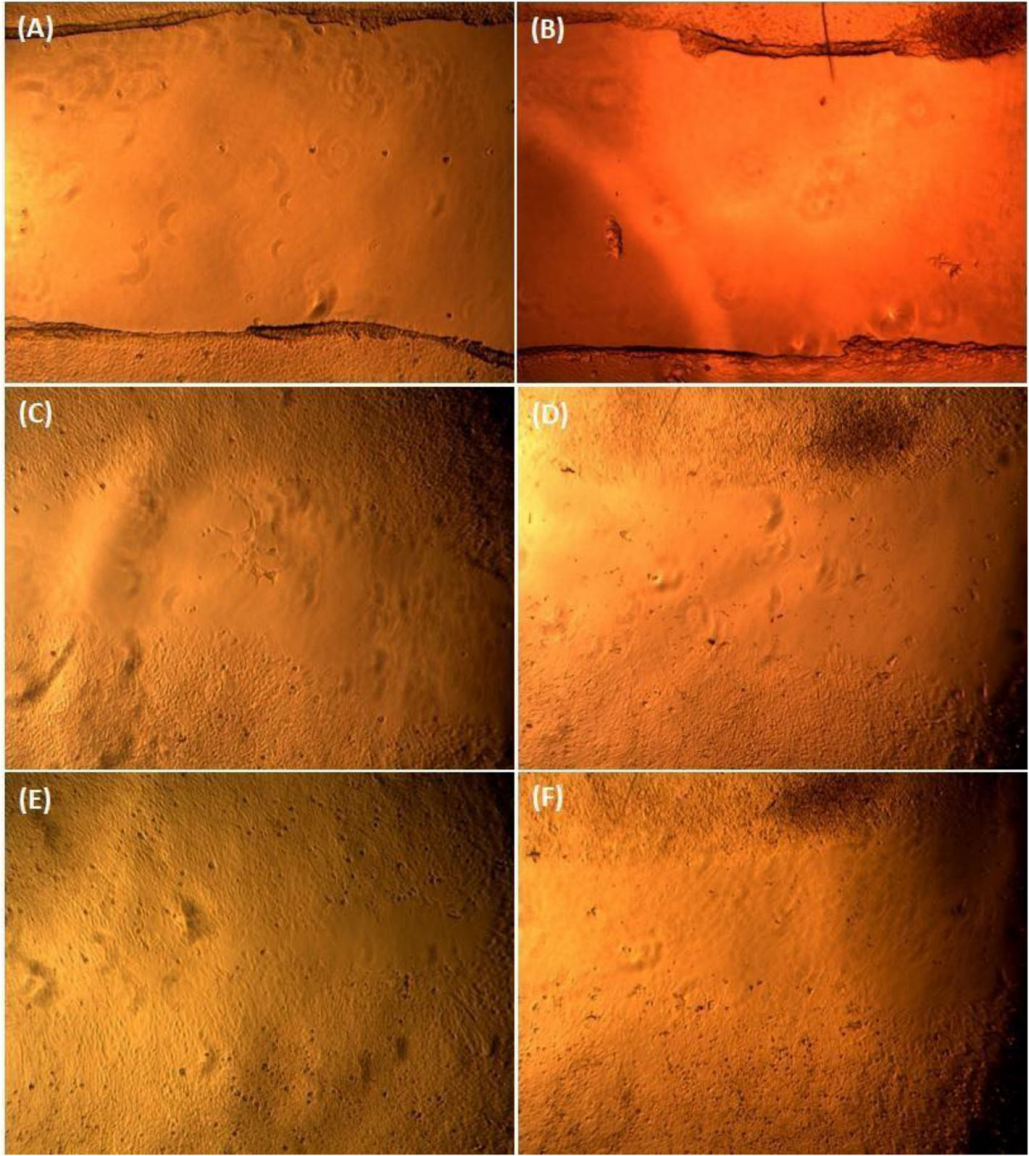
Phase contrast microscopic images of PA 1 cells after 0th hour of incubation. **A** Control, **B** treated 24th hours of incubation, **C** control, **D** treated 48th hours of incubation, **E** control, **F** treated

Migration assay is a hallmark assay to determine the effect of anticancer drugs against angiogenesis and metastasis. PA1 cells were created with wound and were treated with LD 50 value of the *A. vasica* extract. The cells were further observed for 0 h, 24 h, and 48 h against an untreated control. The observation implicated that the cells treated with the *A. vasica* extract did not show any growth in the clear area even after 48 h of incubation, whereas the untreated control was observed with almost complete growth in the clear area, which distinctly reveals that the *A. vasica* extract strongly inhibited the metastatic property in treated PA1 cells (Fig. 4).

### Gene expression studies

**Fig. 5:**
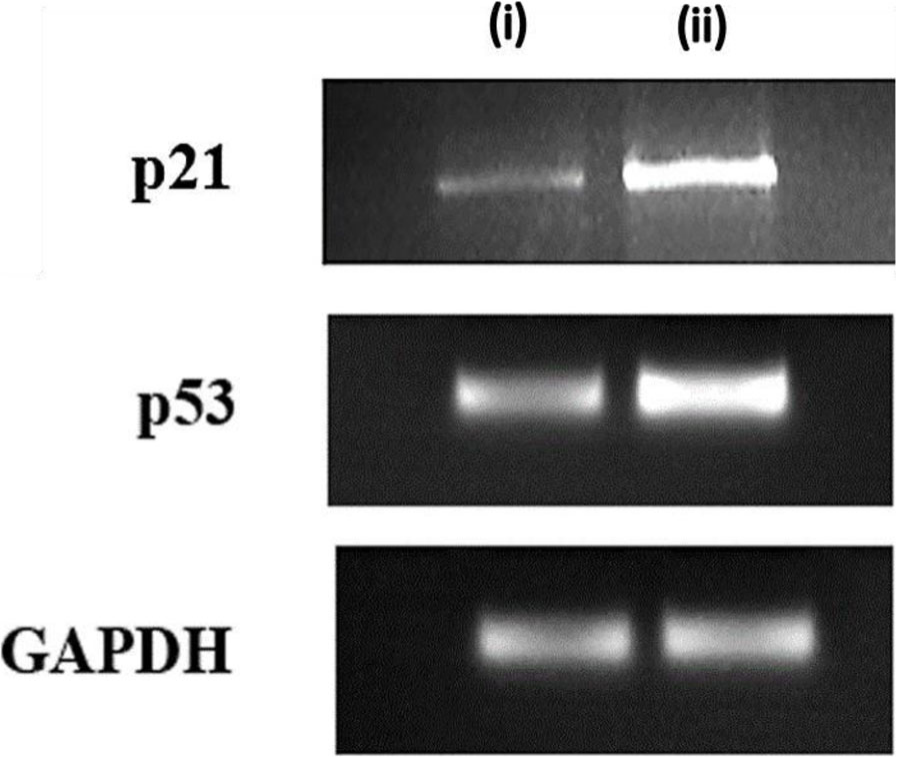
Gene expression analysis of p21, p53, and GAPDH genes in (i) control cells and (ii) treated cells

**Fig. 6:**
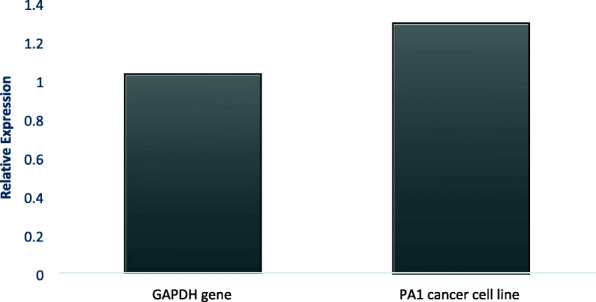
Relative expression of p53 compared to GAPDH gene on the control and treated sample of PA1 cancer cell line

**Fig. 7:**
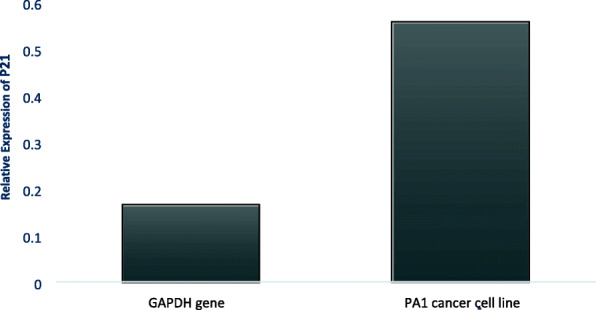
Relative expression of p21 compared to GAPDH gene on the control and treated sample of PA1 cancer cell line

Gene expression studies were conducted in mRNA isolated from the cells treated with the *A. vasica* extract, which were further converted to cDNA and were amplified for the analysis of the p53 gene, p21 gene, and GAPDH gene expression (Fig. 5). Figure 5 demonstrates the changes in the expression of p53 and p21 in comparison to the housekeeping gene (GAPDH). Elaboration of it is provided in Figs. 6 and 7 by comparing the expression of both the tumor suppressor genes (p53 and p21) between the untreated and extract-treated cells.

Both p53 and p21 genes showed increased expression in treated cells when compared to untreated control which indicated that the *A. vasica* extract induced an increase in expression of both genes in PA 1 cancer cell lines which was confirmed by amplifying GAPDH genes which had comparatively same intensity in both treated cells and untreated control (Figs. 6 and 7).

## Discussion

Among gynecologic malignancies, ovarian cancer is the most lethal and the cellular origin of EOC is not well known. Early theories hypothesized that EOC arises from OSE cells, while more recent ones have proposed that it should no longer be considered as a single disease entity but rather a diverse group of tumors with specific morphologic and genetic characteristics [21].

Cancer chemoprevention with natural phytochemical compounds is an emerging strategy to prevent, impede, delay, or cure malignant diseases [22, 23]. *A. vasica* Nees (Acanthaceace) is a well-known medicinal plant, and its diverse medicinal activities include cardiovascular protection, abortifacient, antitubercular, antimutagenic, antiulcer, antiasthmatic activities, hepatoprotective, antibacterial, and anticancer activities [24]. Ethanolic extracts of *A. vasica* were screened for the presence of alkaloids reported to be responsible for diverse biological activities [25, 26].

The ovarian teratocarcinoma cell line PA-1 demonstrated a single chromosomal aberration and is reported to be a useful model cell line used to demonstrate the anticancer efficacy of the investigated agent in in vitro studies [27–29]. Our results clearly showed a decrease in cell viability as per the method of SRB with an LC 50 value of 107.33 μg/ml. The SRB assay has been used since its development in 1990 [30] to inexpensively conduct various screening assays to investigate cytotoxicity in cell-based studies [31]. This method relies on the property of SRB, which binds stoichiometrically to proteins under mild acidic conditions which could be extracted using basic conditions; thus, the amount of bound dye can be used as a proxy for cell mass, which can be extrapolated to measure cell proliferation; hence, EAV is an efficient maneuver to measure the proliferation path of PA1 cell line along with considerable morphological changes similar to that of apoptosis [32].

The study of cell migration in cancer research is of particular interest as the main cause of death in cancer patients is related to metastatic progression. In order for cancer to spread and disseminate throughout the body, cancer cells must migrate and invade through the extracellular matrix (ECM), into blood circulation, and extravasate to form distant foci [31, 33]. In the current study, we used the cell culture wound closure to determine the antimetastatic properties of EAV. From our results, we observed that when compared with untreated control samples, the migration of PA1 cells is significantly decreased in EAV-treated groups which might indicate the antimetastatic properties of EAV [34, 35].

Reverse transcriptase PCR was done to check the effect of EAV on the expression of p53 and p21 mRNA which is already reported to be crucial in controlling uncontrolled proliferation and metastasis. p53 is a nuclear transcription factor and transactivates numerous target genes involved in the induction of cell cycle arrest and/or apoptosis. Under normal conditions, p53 is expressed at an extremely low level, which is caused by proteasomal degradation mediated largely by RING-finger type E3 ubiquitin protein ligase MDM2 p53 which is a nuclear transcription factor with a pro-apoptotic function. Since over 50% of human cancers carry loss of function mutations in the p53 gene, p53 has been considered to be one of the classical type tumor suppressors and reactivation of p53 can be considered as a therapeutic strategy to combat cancer [12]. Most studies of the mechanisms of p53 action have focused on the transcriptional targets of p53, which mediate p53 functions. p21^WAF1^ represents one such target, which plays important roles in cell growth arrest and senescence. p21 can also suppress cell invasion. Notably, it is reported recently that p21 does not simply serve as a downstream mediator of p53 but cooperates with p53 to suppress cell invasion [36, 37]. From our results, it can be observed that p53 and p21 are activated by treatment with KAv which can be suggested as the major mechanisms of action.

## Conclusion

The continued searching for safer and more effective chemoprevention and treatment is clearly needed for the improvement of the efficiency and to lower the treatment cost for cancer care. In this aspect, targeting cancer with phytochemicals is an area of recent research significance, and the present study aimed to determine the antiproliferative and antimetastatic effects of *A. vasica.* Cold percolation methods of *A. vasica* have given alkaloid-rich fractions which were found to decrease the cell viability of PA1 cells when determined by SRB assay. There were concomitant changes in cell morphology with the concentration of extracts. The antimetastatic activity was confirmed by a cell migration assay. The mRNA expression analysis confirmed the reactivation of p53 and p21 genes upon extracts which were found to have a potent role in the anticancer activity of ethanol extracts of *A. vasica.* Further studies can be conducted in vivo to ascertain the efficiency in animals.

## Data Availability

All data generated or analyzed during this study are included in this published article.

## Abbreviations

KNO_3_: Potassium nitrate
NaHCO_3_: Sodium bicarbonate
Na_2_CO_3_: Sodium carbonate
EDTA: Ethylene diamine tetraacetic acid
DMEM: Dulbecco’s modified Eagle’s medium
FBS: Fetal bovine serum
PBS: Phosphate-buffered saline
TCA: Trichloroacetic acid
SRB: Sulforhodamine B
LD: Lethal dose
TE buffer: Tris-EDTA buffer
dNTP: Deoxynucleotide triphosphate
DMSO: Dimethyl sulfoxide
EOC: Epithelial ovarian cancer
